# Single-Cell Tracking of A549 Lung Cancer Cells Exposed to a Marine Toxin Reveals Correlations in Pedigree Tree Profiles

**DOI:** 10.3389/fonc.2018.00260

**Published:** 2018-07-04

**Authors:** Mónica Suárez Korsnes, Reinert Korsnes

**Affiliations:** ^1^Department of Chemistry, Biotechnology and Food Science, Norwegian University of Life Sciences (NMBU), Ås, Norway; ^2^Nofima AS, Ås, Norway; ^3^Korsnes Biocomputing (KoBio), Ås, Norway; ^4^Norwegian Defence Research Establishment (FFI), Kjeller, Norway; ^5^Norwegian Institute of Bioeconomy Research (NIBIO), Ås, Norway

**Keywords:** single-cell tracking, pedigree tree profiles, correlation sister cells, yessotoxin, cancer, epigenetic inheritance

## Abstract

Long-term video-based tracking of single A549 lung cancer cells exposed to three different concentrations of the marine toxin yessotoxin (YTX) reveals significant variation in cytotoxicity, and it confirms the potential genotoxic effects of this toxin. Tracking of single cells subject to various toxic exposure, constitutes a conceptually simple approach to elucidate lineage correlations and sub-populations which are masked in cell bulk analyses. The toxic exposure can here be considered as probing a cell population for properties and change which may include long-term adaptation to treatments. Ranking of pedigree trees according to a measure of “size,” provides definition of sub-populations. Following single cells through generations indicates that signaling cascades and experience of mother cells can pass to their descendants. Epigenetic factors and signaling downstream lineages may enhance differences between cells and partly explain observed heterogeneity in a population. Signaling downstream lineages can potentially link a variety of observations of cells making resulting data more suitable for computerized treatment. YTX exposure of A549 cells tends to cause two main visually distinguishable classes of cell death modalities (“apoptotic-like” and “necrotic-like”) with approximately equal frequency. This special property of YTX enables estimation of correlation between cell death modalities for sister cells indicating impact downstream lineages. Hence, cellular responses and adaptation to treatments might be better described in terms of effects on pedigree trees rather than considering cells as independent entities.

## 1 Introduction

Live cell time-lapse microscopy can be a valuable tool for early diagnosis in cancer therapy. Continuous single-cell tracking over many cell divisions is essential to discover rare cell populations and heterogeneous cell responses, which can be missed in cell bulk assays. It can therefore provide the temporal information that is required to identify differential cell responses and cell fates (1, 2). The main intention of the present work is to contribute to the development of such tools via a case study of tracking individual A549 lung cancer cells exposed to the small molecule compound yessotoxin (YTX). This toxin can induce different cell death modalities in many cellular systems (3, 4). It can activate both caspase-dependent and -independent death pathways (5–12). It also induces different cell death modalities in A549 cells and which fall into two main morphologically distinguishable classes with approximately the same frequency of occurrence. This gives a unique opportunity to observe correlation of cell death modalities for sister cells indicating lineage downstream signaling. This possible communication channel may be significant for enhancement of differences between lineages and adaptation.

YTX can trigger a broad spectrum of cytotoxic responses (13–21). It can also cause anti-allergic and anti-tumoral effects (22) and Korsnes and Korsnes (23) demonstrated its ability to induce genotoxic effects in BC3H1 cells. Several authors have proposed YTX for biotechnological, pharmaceutical, and therapeutic applications due its various cytotoxic and genotoxic effects (4, 24–27).

This work corroborates the capacity of YTX to induce genotoxic effects in A549 lung cancer cells. Treatment of the cells with three different concentrations of this toxin enables to determine variation in individual cell response and cell fate profiles. Cells exposed to YTX are able to carry out abnormal cell divisions affecting cell proliferation. Pedigree profiles evidence how YTX exposure notably affects cell division depending on concentration of the toxin. Asymmetric distributions of the cytoplasm, multipolar divisions, and nuclear changes also confirm this fact. These traits are prominent characteristics of mitotic catastrophe, which is a regulated oncosuppressive mechanism that impedes cell proliferation and/or cell survival owing to extensive DNA damage, problems with the mitotic machinery, and/or failure of mitotic checkpoints (28). It can result from high levels of DNA replication stress or it is caused by an aberrant ploidy or by deregulated chromosome segregation (29, 30).

Single-cell tracking is a developing technology with prospective valuable applications in cancer research and medicine (2). The present work illustrates examples of distinct statistical structures in data from such tracking. Extraction of structures in spatial and temporal observations of single cells can contribute to the development of automatic search for “signatures” of predictive value in large sets of video data. This can help to understand cellular processes and also help timely diagnosis and monitoring for change detection. The approach may be specially relevant for cancer treatment since populations of cancer cells typically exhibit significant variation, and they adapt or become resistant to drug treatments (2, 31–33). Further development of technology for single-cell tracking may include introduction of hardware for new bio-probes to increase the possibilities to monitor intra-cellular organelles and to identify molecular signaling pathways.

## 2 Materials and Methods

### 2.1 Toxin

YTX was obtained from the Cawthron Institute (Nelson, New Zealand). It was dissolved in methanol as a 50 µM stock solution. The stock solution was after diluted in RPMI medium (Lonza, Norway), achieving a final concentration of 2 µM YTX in 0.2% methanol. Treated cells were incubated with 200, 500, and 1,000 nM YTX and control cells were incubated with 0.2% methanol as vehicle. Control cells and treated cells for Hoechst labeling were exposed to different end points 24, 48, 72, and 96 h.

### 2.2 Cell Culture

A549 cell lines were provided by Dr. Yvonne Andersson and Dr. Gunhild Mari Mœlandsmo from the Institute of Cancer Research at the Norwegian Radium Hospital. Cells were cultured in RPMI 1640 (Lonza, Norway), supplemented with 9% heat inactivated fetal calf serum (FCS, Bionordika, Norway), 0.02 M Hepes buffer 1 M in 0.85% NaCl (Cambrex no 0750, #BE17-737G) and 10 ml 1× Glutamax (100×, Gibco #35050-038), 5 ml in 500 ml medium. Cells were maintained at 37°C in a humidified 5% CO_2_ atmosphere.

### 2.3 Time-Lapse Video Microscopy and Single-Cell Tracking

A549 cells were plated onto 96-multiwell black microplates (Greiner Bio-One GmbH, Germany) for time-lapse imaging. Cells were imaged into Cytation 5 Cell Imaging Reader (Biotek, USA), with temperature and gas control set to 37°C and 5% CO_2_ atmosphere, respectively. Sequential imaging of each well was taken using 10× objective. The bright and the phase contrast imaging channel was used for image recording. Two times two partly overlapping images were stitched together to form images of appropriate size. A continuous kinetic procedure was chosen where imaging was carried out with each designated well within an interval of 6 min for a 94 h incubation period. Exposed cells were recorded simultaneously subject to three different concentrations of YTX 200, 500, and 1,000 nM. Control cells were imaged for 26 h in a separate experiment. Technical limitations of the early version of the recording software made it difficult to record all the cells simultaneously because when the density of the control cells became too high, the exposure settings could be compromised. See supplementary data providing video from the recordings.

The single-cell tracking in this work was performed using the experimental computer program *Kobio_Celltrack*.^1^ This system did facilitate to define a rectangle in the middle of the video scene so it initially contained 100 cells to be tracked. Observables from this approach are as follows:

- Pedigree trees where time tagged nodes represent mitosis or cell death and edges stand for observed life span for cells.
- Volume estimates of cells observed to round up before division. These estimates are based on measuring diameters of cells in the state of rounding (short before mitosis).
- Estimates of velocity based on kernel density of positions (Gaussian kernel with fixed bandwidth equal to 15 min).
- Visual classification of cell death.

The visual classification of cell death is assumed to be relatively easy to automatize via image processing.

The present description of heterogeneity in the study cell line includes ranking of pedigree trees based of a measure (“size”) which intuitively represents viability. The common definition of the size of a graph (or tree) *G* is simply its number #*G* of nodes. However, the present definition of size, *M*(*G*), of a pedigree tree modifies this definition as follows:

(1) $$M\left(G\right)\overset{\Delta }{=}\frac{1}{f_{\alpha }\left(\tau \right)}\sum_{c\in G}f_{\alpha }\left(s\left(c\right)\right)$$

where *α* is a tuning parameter (here set to 4 h^−1^) for the function *f_α_*(*x*) = log(*αx* + *e*), *s*(*c*) represents the observed lifetime of cell *c*, and *τ* = 19 h (the doubling time for control cells). *e* is the Euler’s number. Note that an observed lifetime *s*(*c*) counts as 1 if it is equal to the doubling time *τ* simply because $\frac{1}{f_{\alpha }(\tau )}f_{\alpha }\left(x\right)=1$ for *x* = *τ* (cf. Equation 1). The ordering of pedigree trees according to this definition of size *M*(⋅) is only slightly dependent of the value of *α* if it is in the range 1–20 h^−1^.

### 2.4 Nuclear Visualization of Using Hoechst Labeling

1 × 10^4^ control and YTX-treated cells were fixed in 4.0% paraformaldehyde 7.3 pH for 15 min at room temperature. After fixation, cells were washed 3 times with PBS. Cells were incubated with blocking buffer solution (1× PBS in 5% donkey serum and 0.3% Triton X-100) for 15 min. The fixative was removed and then replaced with prewarmed live cell imaging solution containing 50 nM LysoTracker red DND-99 (Life Technologies), and the cells were further incubated for 15 min at 37°C. Cells were washed 3 times with Live cell imaging solution (Termofisher, USA). Two drops of NucBlue^®^ Live ReadyProbes^®^ (Termofisher, USA) was added to a 1 ml live cell imaging solution (Termofisher, USA). The prepared solution was added to the cells and incubated for 7 min at room temperature. Cells were then washed two times with live cell imaging solution (Termofisher, USA). Cells were analyzed with a Leica confocal laser scanner microscope SP5 (Leica Microsystems Wetzlar GmbH, Wetzlar, Germany).

## 3 Results

### 3.1 Revealing Heterogeneity From Single-Cell Tracking

Visualization of pedigree trees from single-cell tracking can help to reveal heterogeneity among cells in a population. It supports detection of possible correlations among mother and daughter cells and between sister cells and which indicates various forms of inheritance from mother to daughter cell. The pedigree trees from the present tracking of A549 cells exposed to yessotoxin, indicate an information transfer downstream pedigree trees and which depends on concentration of the toxin. An example of such inheritance is that sister cells tend to die by similar cell death modality. Information transfer downstream pedigree trees can have interest for assessments on how toxins may affect cells over time.

**Figure 1:**
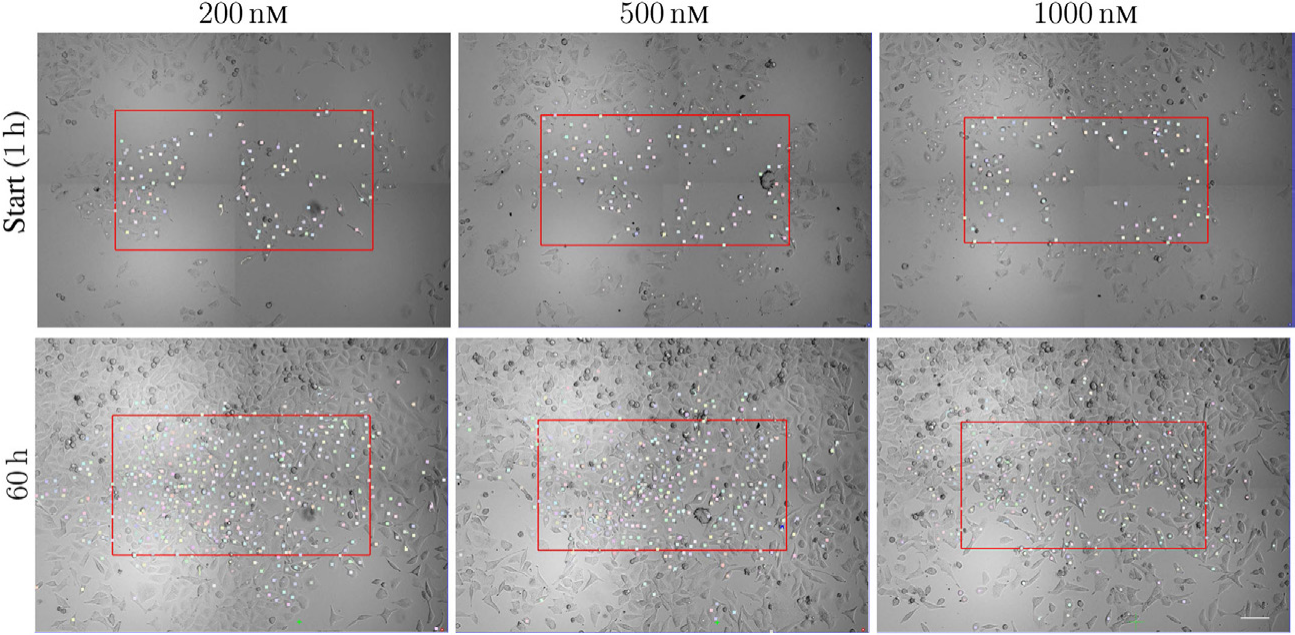
Sample images from time-lapse recording for single-cell tracking. The red frame is large enough to cover initially 100 cells with descendants inside the imaged area during the following time of recording. The frames are of size 888 × 484 μm^2^, 858 × 452 μm^2^, and 840 × 434 μm^2^ (respectively from left to right). The cells are exposed to 200, 500, and 1,000 nM. The lower row shows the cell population at 60 h from the start of exposure. Note the increase of the cell populations from the start to 60 h (largest increase for cells exposed to 200 nM). Many cells move out of the initial red frame during the actual period. Scale bar: 100 µm.

Figure 1 illustrates the organization of the above-mentioned tracking of A549 cells. The figure shows images of the cells after exposure to the three different concentrations 200, 500, and 1,000 nM of YTX during 1 and 60 h. The red frames are here precisely large enough to contain 100 cells at start and which below are called *initial populations*. Five, four, and one of the cells exposed to 200, 500, and 1,000 nM, respectively, had a descendant which left the imaged area (these cells and their descendants were excluded from the statistical treatment below). The supplementary data include video illustrations of the single-cell tracking as well as the pedigree trees resulting from it.

**Figure 2:**
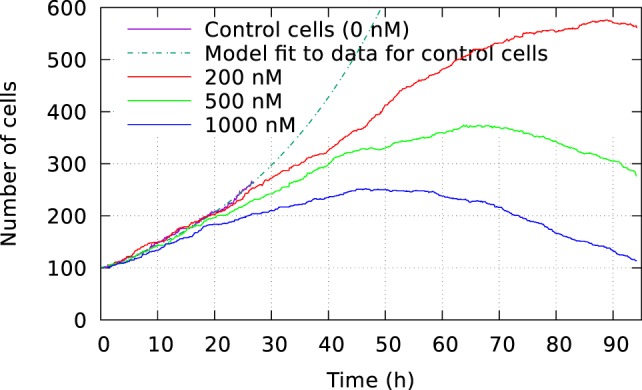
Development of cell size population initially consisting of 100 individuals. The cells were exposed to three different YTX concentrations 200, 500, and 1,000 nM. These data result from tracking the cells which may divide or die. A short recording of control cells indicate initial exponential growth with a doubling time 19 h. Note that the development of population size strongly depends on concentration of the toxin which starts to take effect short after exposure. Subsequent results below show large variations in the development for subsets of these three populations.

**Figure 3:**
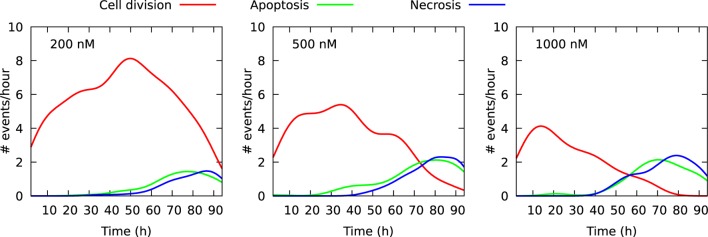
Frequency of cell division and cell death in three cell populations of initial size 100 individuals. The cells were exposed to YTX at concentrations 200, 500, and 1,000 nM. These data result from individual cell tracking. Note that cells start to die at about 40 h after exposure except for 500 nM where apoptosis-like cell death starts to appear after 20 h. Many cells exposed to 1,000 nM enter quiescence after range 30–40 h (see also Figures 6–8 below).

Figure 2 shows the size of these three cell populations as they develop during the observational period of 94 h, whereas Figure 3 shows frequency of cell death in the populations during this period. One can here see that cells start to die mainly after 40 h of YTX exposure. The frequency of mitosis here reduces after 50 h for 200 nM exposure and after 15 h for 1,000 nM treatment.

**Figure 4:**
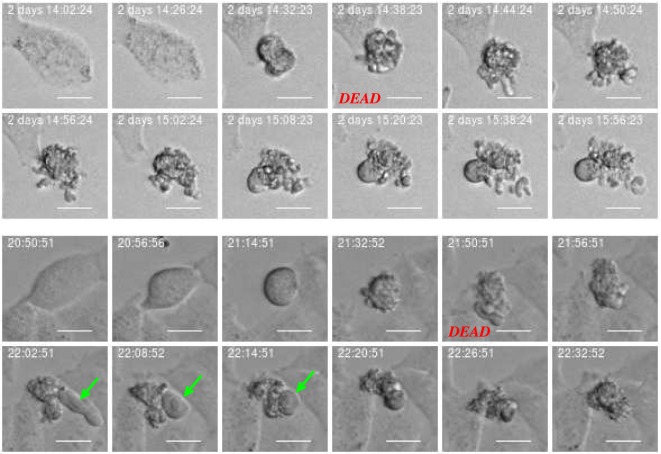
Example of cell death classified as “apoptosis-like” based of imagery recordings. Apoptotic-like cell death evidences cell shrinkage, dynamic membrane blebbing until the cell is systematically dismantled into membrane wrapped vesicles (apoptotic bodies). Green arrow points into apoptotic nuclear disintegration (nuclear extrusion). This morphology can facilitate automatic and objective classification and determination of time for cell death. Scale bar: 20 µm.

**Figure 5:**
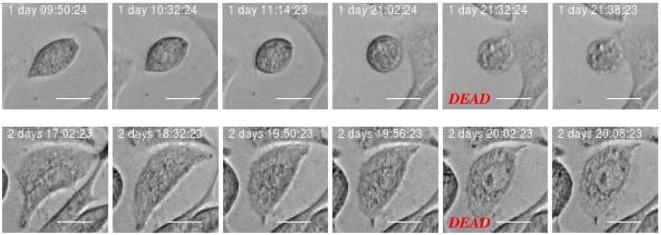
Example of cell death classified as “necrosis-like” based on imagery recordings. Typical features are cytoplasmic granulation and membrane rupture. The necrotic cell looks like fixed/frozen. This morphology can facilitate automatic and objective classification and determination of time for cell death. Scale bar: 20 µm.

The development of the population size reflects the total effect from cell division and death which in this work where visually classified as either apoptosis- or necrosis-like. Figures 4 and 5 clarify this classification based on descriptions of macroscopic morphological alterations as recommended by Galluzzi et al. (28). The classification facilitates automatic classification via computer analysis of image time sequences. Apoptosis exhibits cytoplasmatic shrinkage, plasma membrane blebbing culminating with the formation of apparently small vesicles (apoptotic bodies). Croft et al. (34) suggested that destabilization of the nuclear lamina enables the actomyosin cytoskeleton to tear the nucleus apart and that this process is required to generate apoptotic bodies. Necrosis is morphologically characterized by cytoplasmic granulation, organelle, and/or cellular swelling (oncosis) terminating with membrane rupture (35).

Classification of cell death has historically been based on morphotypes. Its understanding is developing and novel signaling pathways are still being characterized tending to rely on models of signal transduction modules involved in initiation, execution, and propagation of cell death (28). However, the present examples of strong correlation between cell death modalities in daughter cells (see Section 3.2 below) indicates a fundamental biological relevance of the present visual classification.

**Figure 6:**
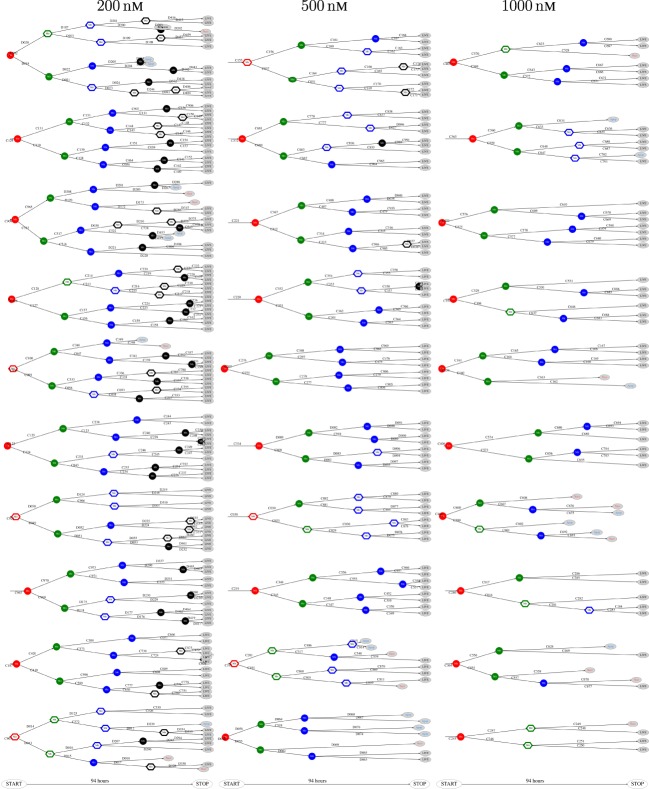
The 10 largest pedigree trees for cells exposed to 200, 500, and 1,000 nM (respectively from left to right). Symbols: “Mit” represents mitosis. Circle here represents normal rounding during cell division whereas hexagon represents no normal rounding. “Apop” and “Necr,” respectively, represents apoptosis and necrosis. “LIVE” means the cell still lives at end of recording.

**Figure 7:**
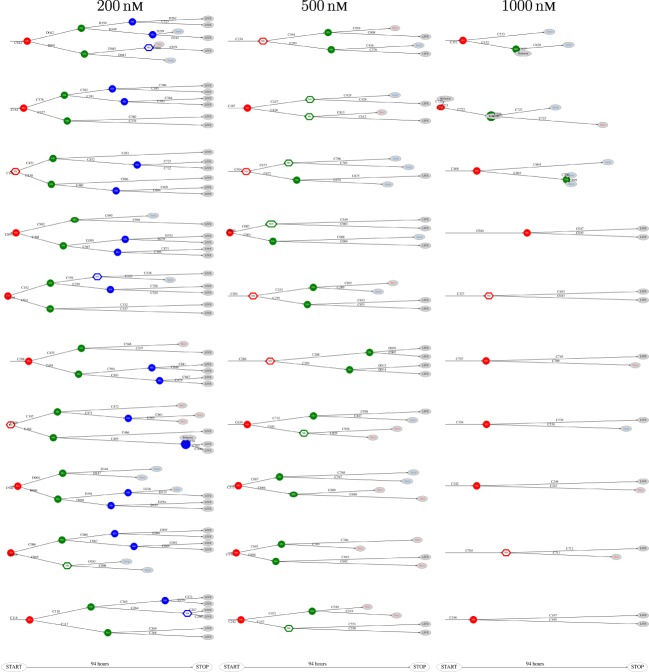
Pedigree tree number 46–55 (“median”) for cells exposed to 200, 500, and 1,000 nM (respectively from left to right).

**Figure 8:**
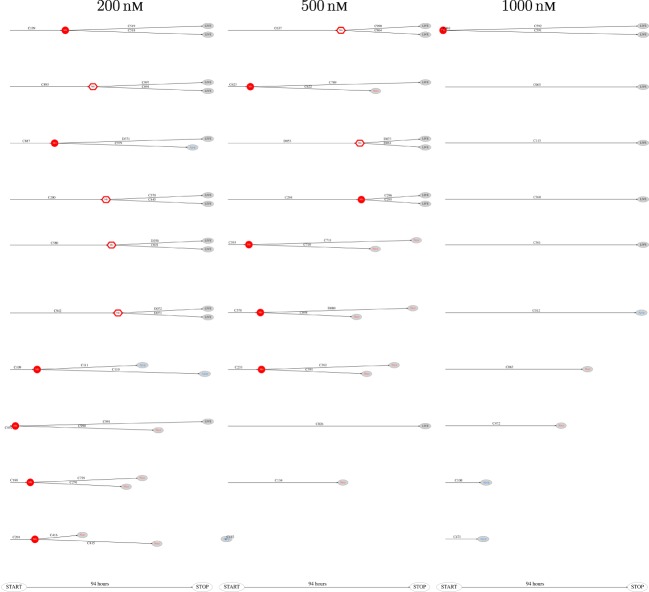
The 10 smallest pedigree trees for cells exposed to YTX at concentrations 200, 500, and 1,000 nM (respectively from left to right).

**Figure 9:**
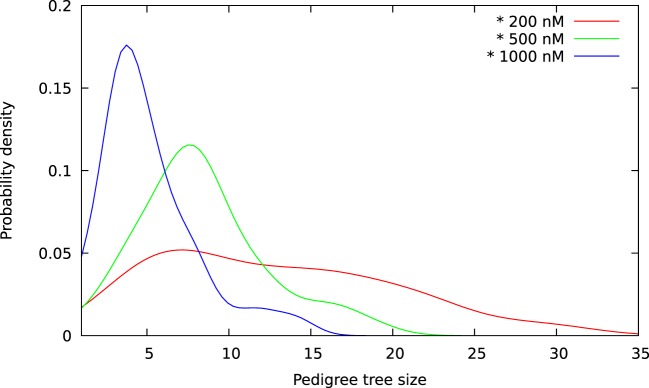
Size distributions for pedigree trees from A549 cells exposed to YTX at concentrations 200, 500, and 1,000 nM. These estimates are kernel densities for the size of three sets of 100 pedigree trees (see text). The kernel bandwidths are here according to the rule of thumb of Silverman (36). Note overlap of size for the three distributions.

**Figure 10:**
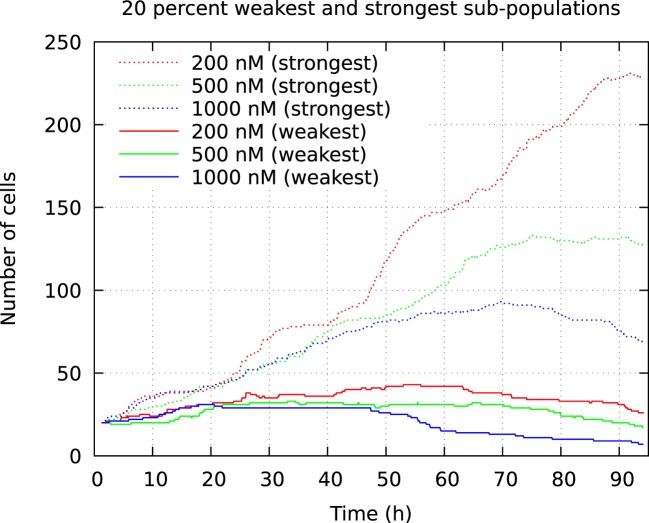
Development of number of cells from the 20% smallest and 20% largest pedigree trees in a cell population initially consisting of 100 individuals. The cells were exposed to YTX at concentrations 200, 500, and 1,000 nM. These data result from tracking the initial cells and their descendants.

The above computer-based single-cell tracking provides pedigree trees where each of the initial cell defines the root of a tree and where events of cell division are time tagged nodes (vertices). A directed connection (arc) between two nodes represents the observed life span of a cell. Figures 6–8, respectively, show the 10 largest (as sorted according to size), the 10 middle (“median”), and the 10 smallest pedigree trees for cells exposed to different concentrations of YTX (200, 500, and 1,000 nM). Equation 1 here defines the (“size”) ranking of pedigree trees. These pedigree trees indicate significant variation of cellular response to the YTX exposure. Figures 9–10 summarize this variability. Figure 9 provides estimates of the size distribution of pedigree trees, and Figure 10 shows the temporal development of number of cells in the 20% largest and the 20% smallest pedigree trees. Figures 6–8 also indicate correlations between cells in the pedigree trees. Assume a sub-tree where a first generation daughter cell is the root node. The size of this sub-tree seems visually positively to correlate with the size of the corresponding sub-tree for the sister cell. The pedigree trees tend in general to appear as somehow symmetric (around the horizontal line through its root). Section 3.2 further elaborates this indication of heritage downstream pedigree trees.

### 3.2 Lineage Inheritance and Information Transfer Downstream Pedigree Trees

**Figure 11:**
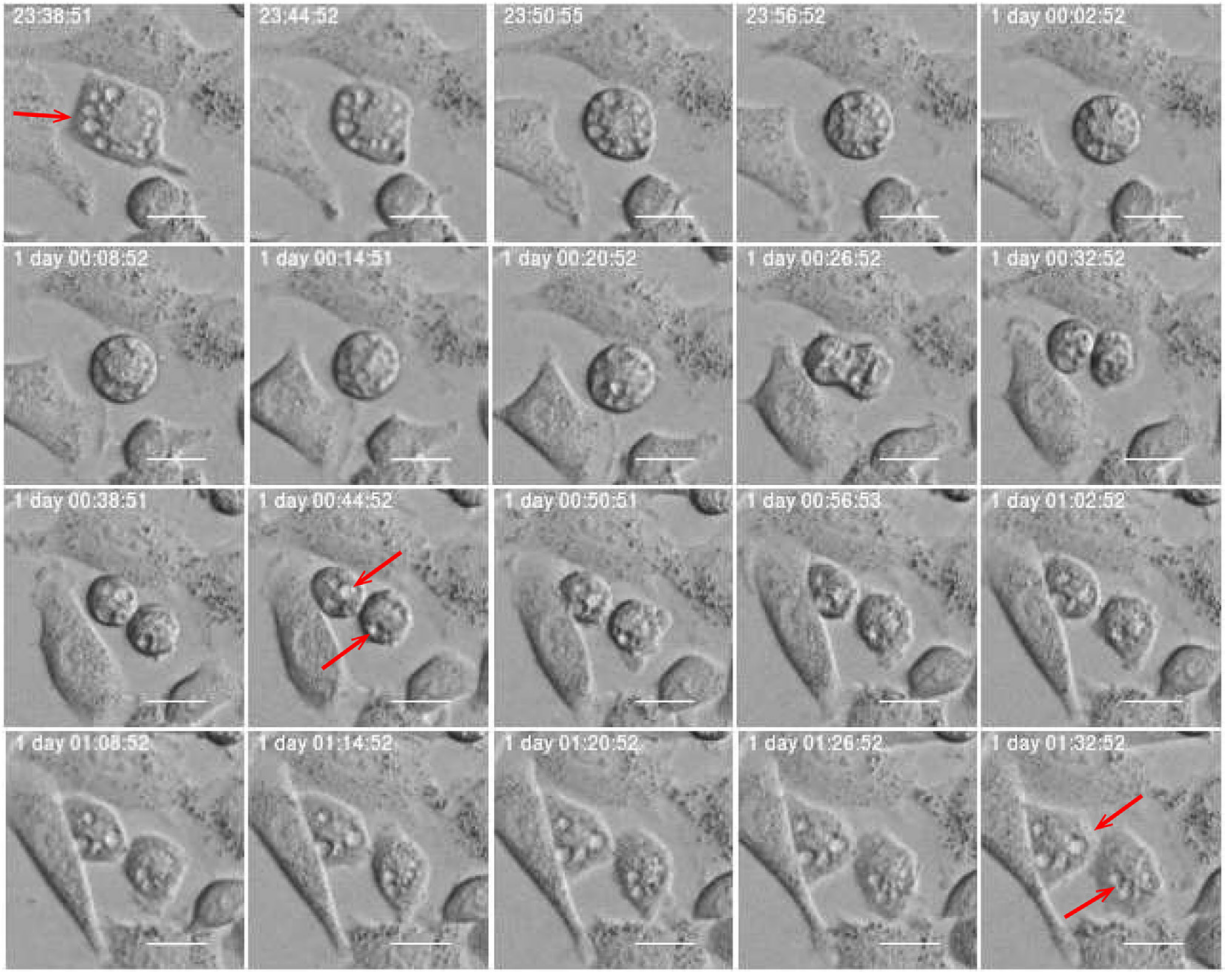
Subsequential images showing vacuole inheritance. Vacuoles (red arrow) pass from mother cell to her daughters through cell division. Scale bar: 20 µm.

**Figure 12:**
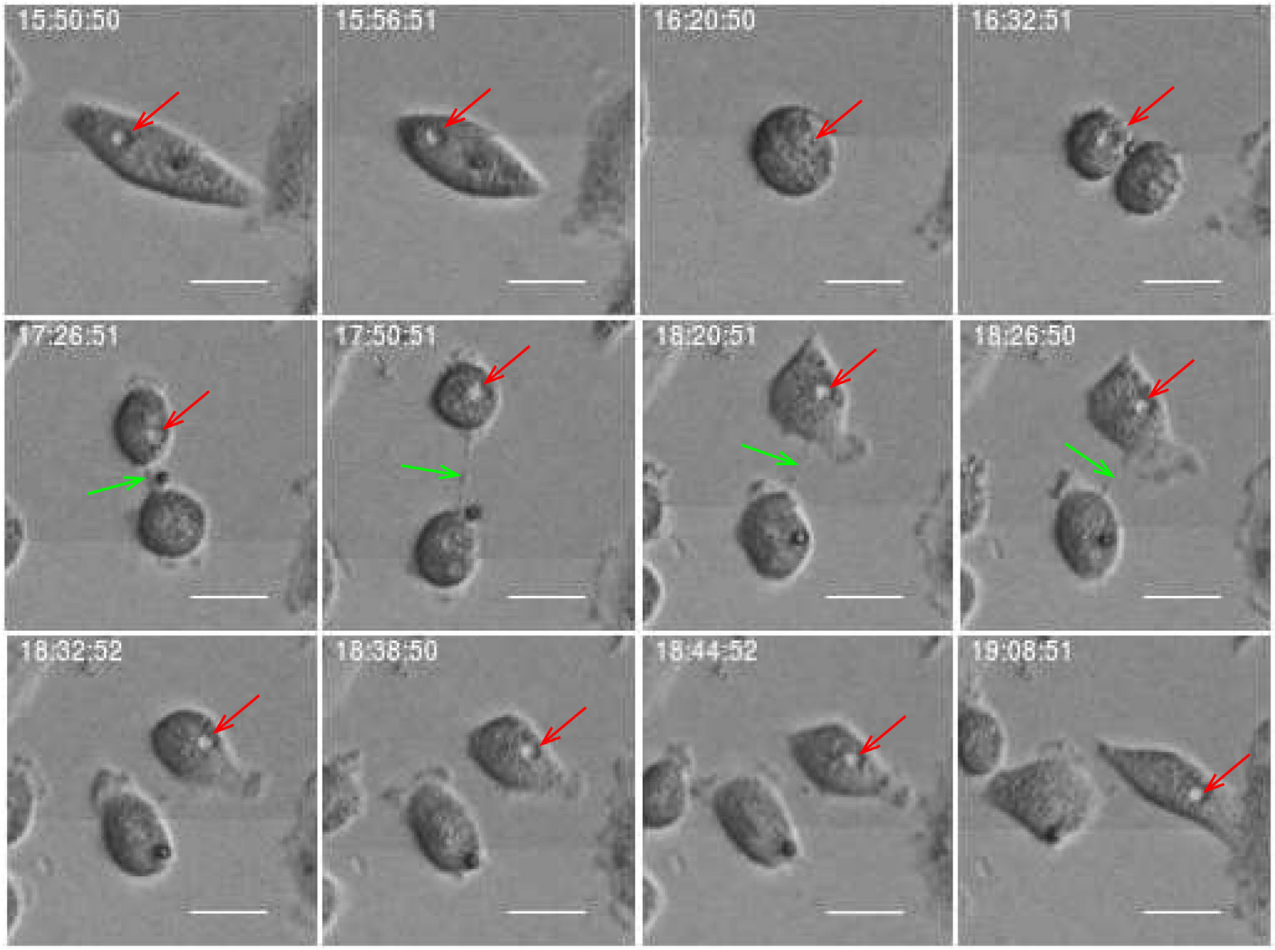
Daughter cell inherits a vacuole from its mother (red arrow). Green arrow points on delay abscission in cells with persistent chromatin in the inter-cellular bridge. Scale bar: 20 µm.

Estimates of correlations between morphological features of sister cells and between mother and daughter cells can contribute to reveal possible inheritance downstream pedigree trees. Figure 11 shows an example where sister cells share morphological features such as vacuoles. It here intuitively looks like the vacuoles of the mother cell are conserved through cell division and transferred to the daughter cells. This may be an indication of the capacity to transfer cell signaling pathways downstream cell division. Vacuoles need time to form, and here they immediately appear after cell division. Hence, it is reasonable to believe that the daughter cells inherited them directly from the mother. Figure 12 more simply illustrates a similar situation. The mother cell here contains one major observable vacuole which one of the daughter apparently inherits from her mother. The size and number of observed vacuoles in the mother and daughter cells are, for both Figures 11 and 12, consistent with the concept that they are transferred through cell division.

It is reasonable to believe that inheritance of other organelles and signal molecules similarly can pass through cell division. Mothers may initiate cell signaling cascades including cell death pathways routing to the daughters since they tend to die in the same way as summarized in Table 1.

**Table 1:** Number of observations of apoptotic- or necrotic-like cell death for sister cells exposed to YTX at concentrations 200, 500, and 1,000 nM.

Apoptosis: A Necrosis: N		Sister 2					
		200 nM		500 nM		1,000 nM	
		A	N	A	N	A	N
**Sister 1**	**A**	12	1	21	5	16	9
	**N**	1	11	3	19	6	22

*This result is from tracking three populations of 100 initial cells, respectively, exposed to YTX at these three concentrations during 94 h. “Sister 1” here denotes the sister with the longest life span*.

The shape of pedigree trees (cf. Figures 6 and 8) gives the impression of correlation between the toxic resistance of sister cells produced by the first (root) cell. The trees seem to have a more symmetric form as compared to hypothetical trees where cell fate where independent for each cell.

**Figure 13:**
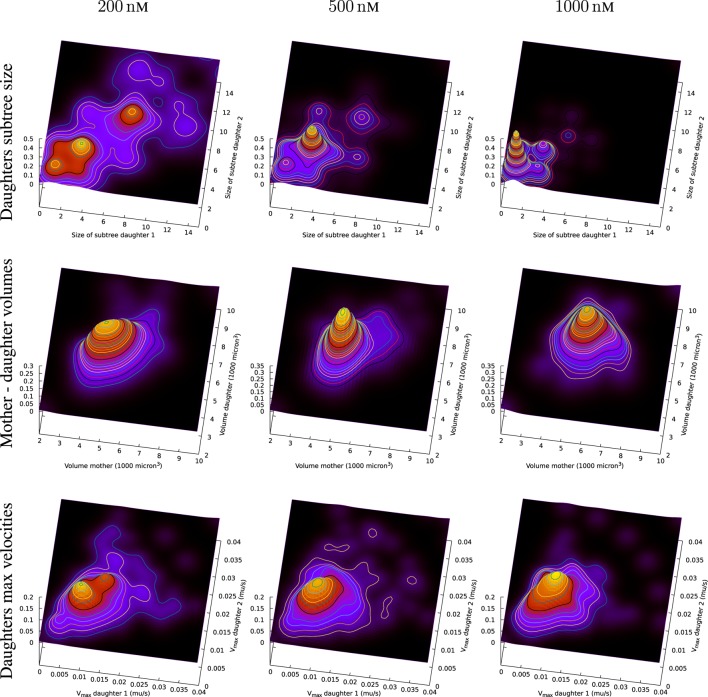
Top row: joint distribution of size of subsequent sub-trees for sister cells. The surfaces are smoothed version of normalized impulses at positions given by associated values from tuples of sister cells. Symmetry is forced by switching values for sisters (artificially doubling the number of observations). Middle row: joint distribution of volume of mother and daughter cell. Bottom row: observed maximum velocity of sister cells.

Figure 13 (upper part) includes an illustration of the correlation between the size of subsequent sub-trees of the first generation of sister cells in the present observed pedigree trees (cf. Figures 6 and 8). The figure shows estimates of the joint probability distribution *p*(*x*,*y*) for the size of the observed pedigree (sub-)trees consisting of the descendants of the first generation sister cells (i.e., the sister cells produced after the first observed cell division of the original pedigree trees).

The estimates of the joint distribution *p*(*x*,*y*) of the size of first generation sister cell sub-trees are kernel density estimates of *p*(*x*,*y*) based on joint observations (*x_i_, y_i_*) of the size *x_i_* and *y_i_, i* = 1,2,…,*N* of sub-trees for *N* tuples of sister cells. It is here no preference between sister cells so the probability distribution *p*(*x*,*y*) is assumed to be symmetric (i.e., *p*(*x*,*y*) = *p*(*y*,*x*)). The observations are therefore swapped to impose symmetry in the way that if (*x_i_, y_i_*) represents an observation, then also (*y_i_, x_i_*) is also part of the set of (joint) observations.

**Figure 14:**
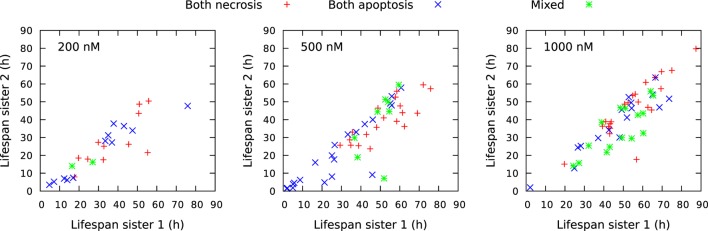
Comparison of life span for sister cells observed to be born and die bye necrosis and/or apoptosis during the observation period of about 94 h. The cells were exposed to YTX at concentrations 200, 500, and 1,000 nM. “Sister 1” denotes the one with the longest life span of two related sisters. Note that there are more mixed cell death modalities (green) for sister cells exposed to 1,000 nM YTX as compared exposure with the lower concentrations. Necrosis tends partly to appear later than apoptosis for exposure by 500 nM.

Figure 13 shows a rich structure of the joint distributions *p*(*x*,*y*) for 200 nM exposure. The distribution seems to reflect three main groups of pedigree trees reflecting different toxic resistance. These groups also seem to match main parts of the distribution for 500 and 1,000 nM exposure. Figure 13 also shows correlations between volume of mother and daughter cells and maximum velocity of sister cells (cf. Section 2.3). A general impression from Figure 13 is that the lowest concentration of exposure tend to give the highest correlations between sister cells and between mother and daughter cells. Table 1 also shows correlation between the type of cell death of sister cells in situations where both sisters are observed to die. Figure 14 shows observed life span for these cells.

The classification in apoptotic- and necrotic-like cell death are here as above (cf. Figures 4 and 5). The table shows that cell death tend to appear as either apoptotic or necrotic for both sister cells exposed to YTX at concentrations 200, 500, and 1,000 nM. Simple statistical hypothesis tests show (for example, by simulation) that the two cell death modalities are clearly correlated. The following test statistic can here serve for formal hypothesis testing independently for each concentration of YTX:

(2) $$z=\frac{N_{different}}{N_{total}}$$

where *N_total_* denotes the number of combined observations of cell death type of two sister cells (“Sister 1” and “Sister 2”), and *N_different_* denotes the subset of observations where cell death modalities are different. Note that there is consistence between the present observations of sister cell death for the three different concentrations of YTX.

### 3.3 Special Sign of Genotoxicity

**Figure 15:**
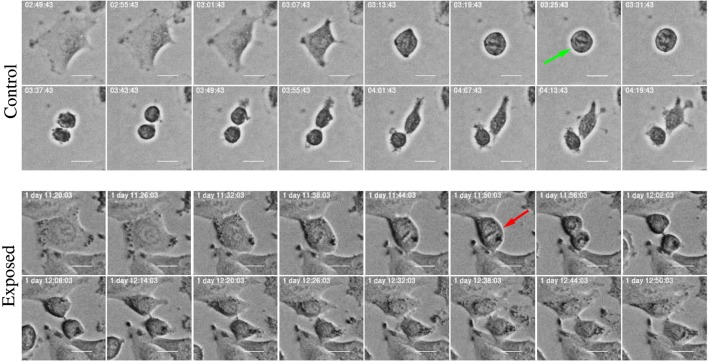
Time-lapse images of mitosis in control and exposed cells treated with YTX. Control cells exhibit normal mitotic rounding and the cells adopt a complete spherical form (green arrow) indispensable for timely mitotic progression. Exposed cells show failure in cell rounding (red arrow) which may induce defects in spindle assembly, pole splitting, and delay in mitotic progression. Scale bar: 20 µm.

**Figure 16:**
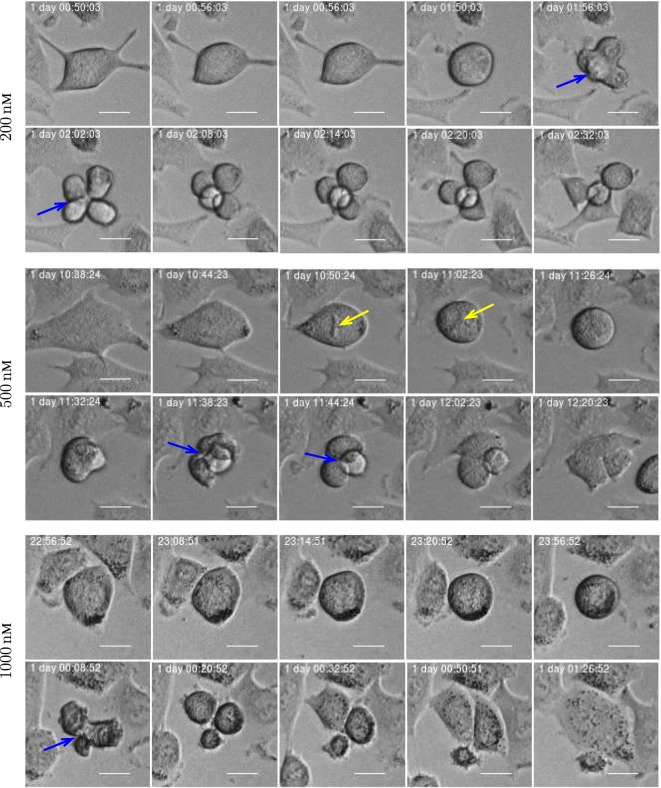
Three examples of asymmetric cell division for A549 cells exposed to YTX at concentrations 200, 500, and 1,000 nM (respectively from top to bottom). Blue arrow illustrates multipolar mitosis and yellow arrow shows a defective mitotic spindle morphology which may affect chromosome alignment. Scale bar: 20 µm.

**Figure 17:**
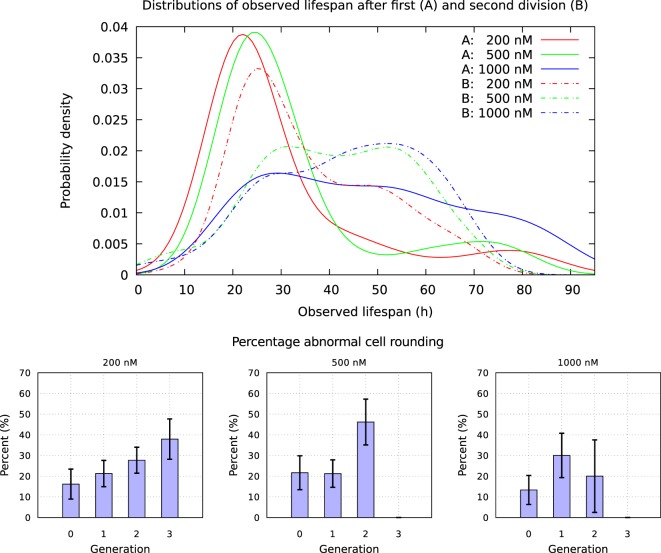
Upper part: distribution of observed life span for cells exposed to YTX at concentrations 200, 500, and 1,000 nM after first and second division. Lower part: fraction of mitosis events without proper cellular rounding. Note that this fraction roughly seems to increase for each cell cycle. A cell is here formally defined to “round up” if the radius of the maximum disk included in/inside (the image of) the cell and the radius of minimum disk including/covering the cell, differ less than 10%.

**Figure 18:**
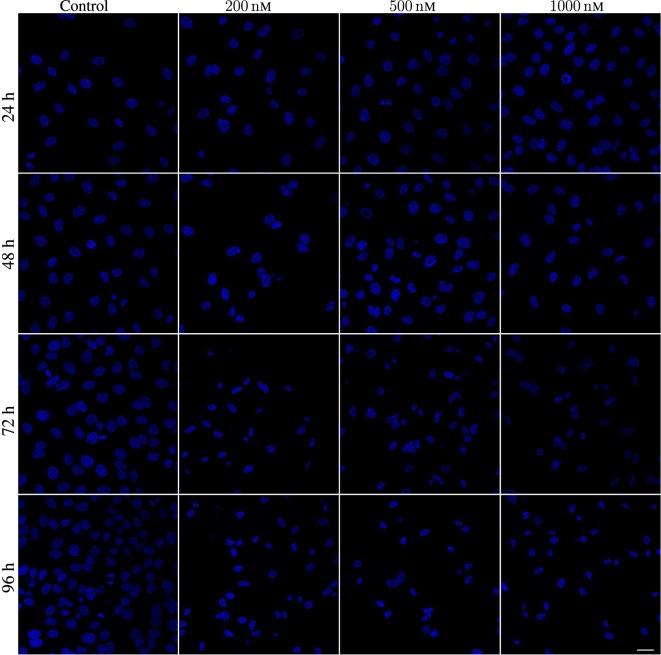
Hoechst labeling of A549 cells showing nuclear envelope defects after being exposed to 200, 500, and 1,000 nM YTX for 24, 48, 72, and 96 h. First column: Hoechst labeling for control cells showing normal nuclei with normal nuclear envelopes. Second, third, and fourth columns show cells exposed to 200, 500, and 1,000 nM, respectively. Note deformed nuclei with lobulated nuclear envelopes in YTX-treated cells. Scale bar: 25 µm.

A549 cells exposed to YTX often exhibit various types of abnormalities during mitosis, delay in mitotic rounding, abnormal midbody structure which is usually thick or very elongated between diving cells, delay in resolution of chromatin bridges which may contribute to failure in cytokinesis (cf. Figures 12, 15 and 16). Failure in cytokinesis can lead to multipolar mitosis and asymmetric cell divisions (29, 37–40). YTX exposure tends to make A549 cells to delay a second round of mitosis. Korsnes and Korsnes (23) showed a similar effect on BC3H1 cells and which indicates genotoxicity. Figure 17 shows the distribution of observed life span of cells after the first and second cell division. Note here that only a part of the population tend to delay the second round of division or die. This means that some cells seem to resist the toxin treatment much better than others. Figure 17 (lower part) also shows that the frequency of abnormal cell rounding increases downstream pedigree trees (and later in time). This additionally supports the idea that YTX is genotoxic for A549 cells. Results from Hoechst labeling (Figure 18) also support it. Such labeling reveals nuclear shrinkage and nuclear envelop deformation adopting a lobulated form. These are typical signs of genotoxic effects.

## 4 Discussion

Tracking of single A549 cells exposed to YTX reveals heterogeneity and lineage correlations in cell response depending on the concentration of the toxin. Korsnes (4) brought up the possibility to use YTX as a tool to induce different cell death modalities, and she demonstrated this potential exposing BC3H1 cells to 100 nM YTX. The present selection of YTX concentrations (200, 500, and 1,000 nM) cause induction of “apoptosis-like” and “necrosis-like” cell death to occur with about the same frequency for A549 cells. These concentrations did also practically help to reveal how pedigree tress can depend on concentration. The unique capacity of YTX to trigger different cell death modalities at approximately the same frequency (for the present range of concentrations), enables to correlate these modalities for sister cells. The observed tendency of sister cells to die the same way, indicates a general “channel” or capacity for downstream signaling and adaptation to stress. This can be a mechanism for accumulation of epigenetic memory. Such accumulation may partly explain the observed heterogeneity among the cells. However, genetic variation can also contribute to it.

A further development of the present study may include comparison of populations with slightly different genetic composition. The comparison may reveal to which extent such differences can affect the statistical properties of pedigree trees. Long-term cultivation of cells under slightly unlike conditions is a conceptually simple way to produce different populations for such experiments.

The possibility to test hypotheses against observations generally makes them more interesting than otherwise. Single-cell DNA sequencing may in different ways provide testing of the conjecture that epigenetic factors are significant to explain the observed heterogeneity among A549 cells exposed to YTX. Assume exposed cells are tracked for a period long enough to form pedigree trees of various sizes. Then the tracking may be stopped for subsequent sequencing in a way so single cells still can be identified as part of a pedigree tree. This enables to correlate the DNA of single cells with their life history. A complementary approach using DNA sequencing is to make “twin studies” of sister cells or make analyses of subsequent pedigree trees for them. The results above (Section 3.2) show that sister cells are correlated with respect to how that die or the viability of their descendants (size of the pedigree tree formed by their descendants). Assume one manage to retrieve many single cells for DNA sequencing, but still let many of then continue undisturbed. Also assume one manage to conserve the identity of all cells (those retrieved and those not retrieved). Then one may know (statistically or partly) the potential fate of individual sequenced cells as if they are still alive. This approach, however, technically challenging, could contribute to distinguish between a hypothesis that DNA mostly counts for heterogeneity as opposed to the possibility that observed variation depends on epigenetic mechanisms.

Single-cell tracking directly indicates that yessotoxin is genotoxic for A549 cells. Korsnes and Korsnes (23) showed similar effects for BC3H1 cells. A sign of genotoxicity for cells exposed to a toxin is that they tend to exhibit aberrant mitosis and multipolar divisions (29, 41–44).

Dividing cells normally adopt a short-term spherical shape known as mitotic cell rounding (45). This behavior is common for most eukaryotic cells ensuring that all chromosomes are timely captured by bringing them close together with spindle microtubules (45). Hence, proper mitotic rounding is considered to enable efficient and stable bipolar spindle assembly for precise and timely mitotic progression (46).

YTX treatment can affect mitotic rounding of A549 cells. The cells can fail to reach proper spherical rounding or the rounding takes long time. This may disrupt spindle assembly altering chromosome capture during mitotic progression which may enable asymmetric cell divisions as shown in Figures 15 and 16.

The nuclear pore complex and components of the nuclear envelop can have different active roles in mitotic events (47). Deregulated division of cancer cells are prone to defects in both the morphology and proteins of the nuclear envelop (48). Its possible structural changes such as low levels of lamins can result in lobulated nucleus (49). YTX treatment tends to make the nuclear envelop to adopt lobulated forms. This probably results from alterations in lamin levels or other key structural nuclear envelop proteins. Lamins undergo dramatic remodeling during cell division (50), and errors here can contribute to various alterations including aneuploidy, mitotic spindle assembly, and other profound aberrations in mitosis (51, 52).

Section 3.2 provides rationales for the idea that signaling proteins can transfer directly from a mother cell to her daughters where they play a role in their subsequent fate. The idea to use time-lapse studies to reveal such information transfer downstream cellular lineages is not new. Both Arora et al. (53) and Barr et al. (54) point out the possibility of using time-lapse studies to link information about how endogenous DNA replication stress in mother cells can pass through daughter cells and later generations.

Transfer of information downstream lineages may change cell populations and facilitate accumulation of information including adaptation to toxins. Adaptation can here be looked at as a simple form of learning. An open question is how developed or complex this potential learning may be and if there are evolutionary conserved “channels” for signaling downstream pedigree trees to provide input for “decisions.” The present results indicate that 1,000 nM YTX exposure reduces correlations between cells downstream pedigree trees as compared to the exposure at lower concentrations (200 and 500 nM). One may therefore expect that exposure at higher concentrations reduces the ability to adapt to toxic stress.

Cell lineages may link observations from different cells and help to provide prognoses from combined analyses. Parameters derived from one event of mitosis may statistically correlate to later events, but without knowing about possible related or “linked” events, an event may appear as “random.” Similarly, two events of cell death may appear (unconditionally) independent. However, with the possible information that the cells are sisters, they may be considered dependent (cf. Table 1). The information that cells are sisters, provide additional information useful to predict their possible cell fate. Two events may, in terms of statistical theory, be independent, whereas they are *conditionally* highly related. Kinship relations may therefore serve to link large amounts of observations of cells to find information of interest.

Data from tracking single cells subject to various treatments can be stored in large combined databases to make it available for computerized data mining (such as application of “Big Data”). The treatments of cells may function as probing them for information. Some treatments may also provide information on potential bioactivity of toxins (bioprospecting). Computerized search in data from single-cell tracking can presumably bring knowledge of medical relevance beyond the reach via direct single human assessments. It may produce prognoses and diagnoses best fitting to, for example, clinical observations. The approach may help to find connections between *in vitro, in vivo*, and clinical data and in this way bringing extra value from, for example, experiments on cell lines.

Smart computer systems can in principle find structures in data and optimize definitions to improve predictive power. Structures in lineage data can provide inspiration and also be directly relevant for establishing computerized treatment of data from singe-cell tracking. The definition of, for example, the “size” of a pedigree tree (Equation 1) is here only meant to be a pragmatic attempt to reflect viability according to a simple linear ordering. A computer system may optimize this definition to uncover structures of biological or medical relevance. The present linear ordering of pedigree trees may generalize to relations involving many parameters (not only one as above). The general philosophy here is that preliminary semi-optimal attempts can contribute to find models of more predictive power.

Collections of pedigree trees from cells subject to different treatments can provide quantification of diversity, detection of change in populations, and emergence of sub-populations as well as possible signaling downstream pedigree trees. Large sets of pedigree trees can facilitate automatic search for signatures to find relations and knowledge otherwise not available from limited experiments. Probabilistic descriptions of pedigree trees can give opportunities to track cells in more effective multi-target based ways as compared to a naive approach following singe cells independently one at a time. It can help to resolve ambiguities in cell tracking and in this way facilitate efficient sampling and error detection.

Cancer cells may progressively accumulate genetic mutations derived from clonal evolution. However, only a clonal minority may be responsible for cancer progression (55–57). Epigenetic changes also contribute to cellular heterogeneity because they promote changes in gene functions/interactions and propagate heritable changes in the phenotype without affecting the DNA sequence (56). These changes can maintain the phenotype into the adulthood and for subsequent generations (58, 59).

A type of epigenetic memory which can help adaptation to stress is connected to the nuclear pore complex (NPC) which is a large molecular portal penetrating the nuclear envelop to facilitate nuclear-cytoplasmatic trafficking (60). Guan et al. (61) demonstrated that many yeast genes induced by oxidative stress are activated more rapidly in cells that have previously experienced salt stress. This effect persists for up to four generations after the initial stress.

The significance of epigenetic inheritance of cellular phenotype during cell divisions has remained underestimated (62). The stability of the cellular mRNA and proteins confers the capacity to a cell to conserve a stable gene expression level and transmit it over multiple generations even if transcription and translation are highly fluctuating. In addition, reducing short-term fluctuations through high stability of the molecules can be considered as a simple way of transcription noise reduction at a low energy cost. Indeed, it takes less energy for the cell to maintain the constant level of a protein by not degrading the molecules already present than continuously re-synthesizing them (63).

Several authors have commented on the significance of “non-genetic” information transfer from mother to daughter cells. Memory mechanisms of gene transcription regulation may explain observed transmission of phenotypes downstream lineages (64). However, these mechanisms are also blurred by noise (63) and which may generate variability between cellular lineages.

Genetic mutations and thermal “noise” during protein synthesis may explain variability among treated cells. However, signal transfer downstream lineages (memory) may amplify differences between cells. It is therefore reasonable to believe that if there was a “reset” at each cell division in a clonal population, then there would be less variability than presently observed. The idea that various signal molecules can pass through the mitosis process has general interest since such transfer from mother to daughter cells can probably have an evolutionary advantage in avoiding the cost of adaptation. A tendency of “listen to your mother” can, for example, save energy of signaling and sensing as compared to a full “reset” during mitosis.

The cost saving by avoiding “reset” at cell division may be in terms of energy, risk of failures, and restrictions for different cellular processes. A mother cell might signalize to her daughters not to divide to avoid high transmission of replication errors, however, those cells may still have a function in the organism. Unresolved replication stress inherited from a mother cell may cause her daughters enter quiescence (53). Parental experiences from environmental stress can increase the stress sensitivity of their descendants requiring adjustments of their chromatin structures (65).

A main concern in cancer treatment is development of drug resistance. Random genetic mutations may occasionally make some cancer cells viable even under treatment and which sub-sequentially initiate a resistant sub-population. Another way to drug resistance is that mother cells transfer information to their daughters such as damaged proteins or low-level of DNA damage and which sometimes can increase robustness in a cell population leading to cell proliferation (53, 66). Signals from a mother cell may, for example, help her daughters to save cost establishing counter-measures to toxic exposure such as DNA repair mechanisms. Cell tracking experiments may in principle help to distinguish between these two hypotheses. Genetic mutations are random events whereas adaptation via signaling downstream pedigree trees is to a larger extent deterministic and would presumably take place in several pedigree trees pretty close in time as opposed to random mutations which would appear as rare singular events.

Single-cell tracking analysis is therefore a powerful approach that provide more precise analysis of rare sub-populations masked in cancer cell populations. Transfer of information between single cells can take place via epigenetic changes and these changes are conserved through descendants. Data analysis derived from single-cell tracking allow elaborating pedigree tree profiles and discover that those profiles may vary significantly applying the same concentration of toxin treatment. This information may be relevant for treatment of cancer drug resistance which is a common characteristic acquired for many types of cancer. New technology for high-resolution observations of molecular signaling pathways is a prospective further step in this development of methods to control cancer.

## Author Contributions

MK conceived the study and conducted the laboratory experiments, RK made the computer programming; both authors analyzed the results and wrote the manuscript.

## Conflict of Interest Statement

The authors declare that the research was conducted in the absence of any commercial or financial relationships that could be construed as a potential conflict of interest. The reviewer HA and the handling Editor declared their shared affiliation.

## Footnotes

^1^https://www.korsnesbiocomputing.no/ (Accessed: June 30, 2018).
